# Population-wise incidence and outcomes of patients requiring invasive and non-invasive mechanical ventilation in China: a nationwide retrospective analysis by age, sex, and comorbidity

**DOI:** 10.1186/s13613-025-01537-w

**Published:** 2025-08-14

**Authors:** Maomao Cao, Rong Liufu, Boyang Wang, Wei Pan, Hongda Chen, Longxiang Su, Yun Long, Xiang Zhou, Li Weng, Bin Du, Critical Care Clinical Trials Group, China National Critical Care Quality Control Center Group

**Affiliations:** 1 Medical Intensive Care Unit, State Key Laboratory of Complex Severe and Rare Diseases, Peking Union Medical College Hospital, Peking Union Medical College & Chinese Academy of Medical Sciences, Beijing, 100730 China; 2 Department of Critical Care Medicine, State Key Laboratory of Complex Severe and Rare Diseases, Peking Union Medical College Hospital, Peking Union Medical College & Chinese Academy of Medical Sciences, Beijing, 100730 China; 3 Center for Prevention and Early Intervention, National Infrastructures for Translational Medicine, Institute of Clinical Medicine, Peking Union Medical College Hospital, Peking Union Medical College & Chinese Academy of Medical Sciences, Beijing, 100730 China; 4 Department of Critical Care Medicine, Peking Union Medical College Hospital, Peking Union Medical College & Chinese Academy of Medical Sciences, Beijing, 100730 China

**Keywords:** Mechanical ventilation, Epidemiological study, Hospitalization, China

## Abstract

**Background:**

Mechanical ventilation is a critical yet labor-intensive medical resource with limited availability. However, population-based data on its utilization and outcomes remain scarce in China. This study aimed to describe the characteristics, frequency, and outcomes of mechanical ventilation at the national level in China.

**Methods:**

In this multicenter cross-sectional study, we retrospectively identified hospitalized patients who received mechanical ventilation using data from the National Data Center for Medical Service. The dataset included information on patient characteristics, length of hospital stays, procedures, diagnoses, and discharge outcomes. The population selected for this study included all mechanically ventilated patients admitted between January 1, 2022 and December 31, 2022. We analyzed the distribution characteristics of patients requiring mechanical ventilation by type of ventilation. Case fatality rates were calculated and further stratified by age, sex, and comorbidity burden.

**Results:**

The study included 1,641,809 admissions from 2,837 hospitals, with all patients receiving mechanical ventilation. The median age of the patients was 66.0 years (interquartile range: 51.0–76.0). Among them, 64.4% received invasive mechanical ventilation (IMV) only, while 29.0% received non-invasive ventilation (NIV) only. The incidence of mechanical ventilation was 186.5 per 100,000 population. Patients receiving IMV only had longer hospital stays and a higher comorbidity burden, compared with those receiving NIV. 41.4% of invasively ventilated patients had a diagnosis of cerebrovascular disease. In contrast, chronic pulmonary disease was the most common comorbidity (57.0%) in NIV patients. The NIV failure rate observed in our study was 7.9%. Overall, 12.8% of mechanically ventilated patients died during hospitalization, with a marked difference in case fatality rate between IMV patients (16.2%) and those receiving NIV only (5.5%). Increasing age and a higher Charlson index were both associated with a stepwise increase in mortality risk.

**Conclusions:**

Significant variations in epidemiological characteristics by age, sex, and comorbidity were observed across different modalities of mechanical ventilation. Mortality rates were markedly higher among patients receiving IMV compared to those receiving NIV, with the differences most pronounced among elderly patients, males, and those with greater comorbidity burden.

**Supplementary Information:**

The online version contains supplementary material available at 10.1186/s13613-025-01537-w.

## Background

Mechanical ventilation, a critical life-support tool, plays a crucial role in the treatment and management of critically ill patients with conditions such as acute respiratory distress syndrome, sepsis, and pneumonia [1–3]. The substantial healthcare costs associated with mechanical ventilation cause a significant financial burden on patients and healthcare systems [4]. During the COVID-19 outbreak, the global shortage of ventilators highlighted the need for strategic planning in the allocation of mechanical ventilation resources [5]. As the pandemic subsides, understanding the demographic and clinical characteristics of patients requiring different types of mechanical ventilation has emerged as an urgent and essential priority.

Achieving equitable health outcomes through effective policy implementation and resource allocation remains a fundamental global health objective and an ongoing challenge. In this context, large-scale, geographically diverse databases have emerged as valuable tools for analyzing the comprehensive profile and distribution of mechanical ventilation among hospitalized patients at national and international levels [6–8]. For instance, a study conducted in Germany used national administrative claims data to identify mechanically ventilated patients and found that the most common primary diagnoses were cardiovascular disease, pneumonia, and chronic obstructive pulmonary disease [3]. Notably, patients with severe exacerbations of chronic obstructive pulmonary disease treated with non-invasive mechanical ventilation (NIV) had lower mortality rates compared to those requiring invasive mechanical ventilation (IMV) [8]. These findings indicated the importance of understanding potential high-risk patients who are more likely to require different modes of mechanical ventilation, as such insights can inform better clinical decision-making.

China is currently facing an escalating burden of chronic diseases, an aging population, recurring public health emergencies, and infectious outbreaks [9–11]. These challenges highlight the urgent need for large-scale epidemiological data to analyze the distribution patterns of mechanically ventilated patients across the nation. However, research on mechanical ventilation in China, with its distinct characteristics in disease burden and considerable epidemiological disparities, is very scarce. To bridge this gap, we established a nationwide mechanical ventilation database that integrates data from 31 provinces. In this study, we aimed to describe the process of database construction and conduct a nationwide comparative analysis of age, sex, and comorbidities among patients requiring IMV and NIV in China.

## Methods

### Study design

This was a retrospective analysis based on data from the National Data Center for Medical Service (NDCMS) claims database.

### Data sources

The NDCMS is responsible for the data collection from multilevel hospitals across China, quality assessment, and data summarization. By the end of 2022, the NDCMS had enrolled a total of 1,923 tertiary and 2,363 secondary hospitals across 31 provinces, municipalities, and autonomous regions, representing 64.1% of all tertiary hospitals and 22.7% of all secondary hospitals in mainland China. Admissions recorded in the NDCMS from tertiary and secondary hospitals accounted for 45.0% of all hospital admissions nationwide and 69.6% of all admissions in public hospitals [12]. Data for mechanical ventilation in mainland China across 31 provinces were collected from the NDCMS retrospectively.

The database contains detailed, individual-level information recorded during hospitalization. It includes data such as demographic details, admission and discharge dates, diagnoses and procedures, discharge status, as well as associated healthcare costs. Under data protection principles, the database is securely hosted at Peking Union Medical College Hospital in China and is available for research purposes in an anonymized and secure format. MySQL (version 8.0.41) was used to store structured data in a standardized format. All data were anonymized before use and were divided into domains, including demographic information, treatment records, diagnostic results, and medical costs. Data cleaning and analysis were performed using R software (version 4.3.1) to address logical errors and detect outliers.

### Study population

During the database creation process, we extracted data from the NDCMS claims database to identify patients who required either NIV (International Classification of Disease, Ninth Revision, Clinical Modification [ICD-9-CM] codes: 93.90 or 93.91), IMV (ICD-9-CM codes: 96.70, 96.71, or 96.72) or alternating between both types of mechanical ventilation (i.e., NIV followed by IMV or IMV followed by NIV). NIV failure was defined as the need for intubation after NIV intervention. The study population included all mechanically ventilated patients admitted between January 1, 2022, and December 31, 2022.

Patients were excluded based on the following criteria: those aged < 0 years or > 120 years, those with unknown sex or personal identity, and patients with a length of hospital stay of less than 0 days or greater than 365 days.

### Definition of diseases

Disease diagnoses were coded using the official 10-digit Chinese version of the International Classification of Diseases, Tenth Revision (ICD-10) (an expansion from the 4-digit WHO version), and ICD-9 procedure codes.

### Training and data quality control

The quality of data in the NDCMS was maintained through a multifaceted approach that included a manual review of records, regular quality control meetings, staff training programs, and regulatory inspections. After data collection and entry into the claims database platform, medical record management personnel and coding staff were responsible for assigning disease classification codes and procedure codes. This process was accompanied by hospital-level quality control checks to ensure the accuracy of the information. Subsequently, data were uploaded promptly by information management personnel, following established data transmission interface standards to ensure completeness and accuracy.

To further enhance data accuracy, local validation was performed within the NDCMS by cross-checking local system extractions against the database records. This additional step strengthened the reliability of the data. The dataset boasts a completeness rate exceeding 95%, with demographic, geographic, and diagnosis information achieving 100% completeness.

### Recorded variables

This database provided here includes age at hospital admission (continuous and categorized: 0–6 years, 6–18 years, 18–65 years, 65–80 years, and > 80 years), sex (male or female), geographic region (East, Central, and West), hospital type (secondary hospital or tertiary hospital), length of hospital stay, procedure codes, as well as diagnoses recorded according to the ICD-10, discharge status (survive or death). Previous studies have validated the sensitivity and specificity of codes for diagnosis and procedures [12, 13]. Definitions of all variables within the NDCMS are provided in the Supplementary Table 1 for further reference.

Comorbidities were summarized into the Charlson index using methods described by Charlson ME et al. [14] (Supplementary Table 2). Patients were further grouped according to age groups, the Charlson index, and the type of mechanical ventilation.

### Statistical analysis

Statistical analysis was performed using R software (version 4.3.1). Continuous variables were summarized as either mean ± standard deviation (SD) or median (interquartile range, IQR), as appropriate. Categorical variables were presented as frequencies and percentages. The normality of the data was assessed using the Kolmogorov–Smirnov test. Differences between categorical variables were tested using the Chi-square test. Age, sex, and comorbidity-specific case fatality rates in patients requiring different types of mechanical ventilation were calculated in each stratum as the number of deaths divided by the number of mechanically ventilated patients. The 95% confidence intervals (CIs) were estimated by binomial distribution. A p-value < 0.05 was considered statistically significant.

## Results

**Fig. 1:**
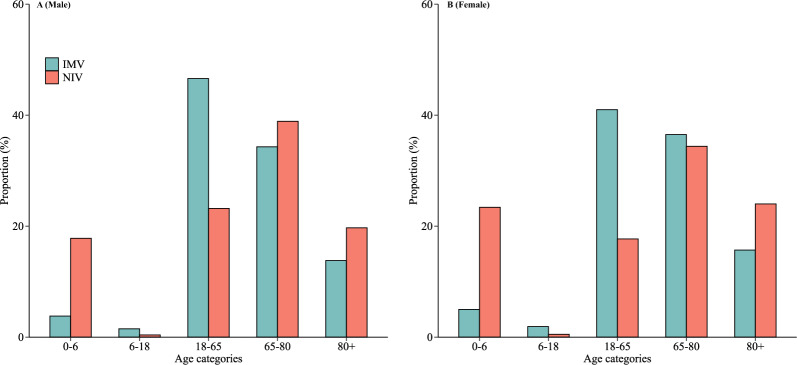
Age distribution of invasive and non-invasive mechanical ventilation by sex

The overall data for mechanically ventilated admissions in China in 2022 are summarized in Table 1. After quality control, a total of 1,641,809 mechanically ventilated admissions were admitted to 2,837 hospitals in China, accounting for 1.6% of all hospital admissions (101,805,967 in total). Of the ventilated patients, the frequency of patients treated exclusively with IMV was 64.4%, whereas 29.0% of patients received NIV exclusively. The overall incidence of mechanical ventilation was 186.5 per 100,000 population. Demographically, patients had a median age of 66.0 years (IQR: 51.0–76.0), and were predominantly male (64.5%). The proportion of patients who were ventilated was 11.6% of those aged less than 18 years, 36.4% of those aged 18–65 years, 35.5% of those aged 65–80 years, and 16.5% of those older than 80. NIV exhibited an age-related pattern, with a marked concentration among infants and older adults, especially those aged over 80 years. This age-related pattern remained consistent even when stratified by sex (Fig. 1). Geographically, 44% of ventilated patients were admitted to hospitals in the Eastern regions. Patients in the Western regions tended to be younger and had shorter hospital stays, compared to those from the Eastern regions (Supplementary Table 3).

**Table 1:** Baseline characteristics according to type of mechanical ventilation

Characteristics	Total population	Type of mechanical ventilation		
		IMV only	NIV only	Both
Number of admissions	1 641 809	1 057 660	476 065	108 084
Number of hospitals, n (%)	2 837	2 797	2 660	2 340
Age, years				
Mean (SD)	59.2 (25.3)	61.2 (20.5)	57.3 (30.9)	47.7 (34.2)
Median (IQR)	66.0 (51.0–76.0)	65.0 (52.0–75.0)	69.0 (50.0–79.0)	63.0 (0.0–75.0)
Age group, years, n (%)				
0–6	171 537 (10.4)	44 254 (4.2)	93 942 (19.7)	33 341 (30.8)
6–18	19 810 (1.2)	17 028 (1.6)	1 930 (0.4)	852 (0.8)
18–65	597 053 (36.4)	471 855 (44.6)	101 434 (21.3)	23 764 (22.0)
65–80	582 889 (35.5)	371 293 (35.1)	177 805 (37.4)	33 791 (31.3)
> 80	270 520 (16.5)	153 230 (14.5)	100 954 (21.2)	16 336 (15.1)
Sex, n (%)				
Male	1 059 546 (64.5)	676 340 (63.9)	313 016 (65.8)	70 190 (64.9)
Female	582 263 (35.5)	381 320 (36.1)	163 049 (34.2)	37 894 (35.1)
Region, n (%)				
East	723 377 (44.0)	488 869 (46.2)	187 322 (39.3)	47 186 (43.7)
Central	400 301 (24.4)	264 184 (25.0)	111 389 (23.4)	24 728 (22.9)
West	518 131 (31.6)	304 607 (28.8)	177 354 (37.3)	36 170 (33.5)
Discharge status, n (%)				
Death	210 480 (12.8)	171 262 (16.2)	26 021 (5.5)	13 197 (12.2)
Survive	1 431 329 (87.2)	886 398 (83.8)	450 044 (94.5)	94 887 (87.8)
Length of hospital stay, days				
Mean (SD)	16.0 (19.4)	16.8 (21.5)	13.0 (12.2)	21.5 (21.8)
Median (IQR)	11.0 (6.0–20.0)	12.0 (4.0–21.0)	10.0 (7.0–15.0)	15.0 (9.0–27.0)
Charlson index, n (%)				
0	412 238 (25.1)	252 780 (23.9)	123 408 (25.9)	36 050 (33.4)
1	367 992 (22.4)	257 323 (24.3)	92 175 (19.4)	18 494 (17.1)
2	294 079 (17.9)	180 762 (17.1)	96 869 (20.3)	16 448 (15.2)
≥ 3	567 500 (34.6)	366 795 (34.7)	163 613 (34.4)	37 092 (34.3)
Initiation reasons, n (%)				
Respiratory failure	517 390 (31.5)	255 205 (24.1)	224 780 (47.2)	37 405 (34.6)
Pneumonia	398 017 (24.2)	218 895 (20.7)	142 202 (29.9)	36 920 (34.2)
Sepsis	280 625 (17.1)	196 724 (18.6)	59 842 (12.6)	24 059 (22.3)
Incidence per 100,000 population	186.5	120.8	55.7	13.5

*IMV* invasive mechanical ventilation, *NIV* non-invasive mechanical ventilation, *SD* standard deviation, *IQR* Interquartile range. To calculate the incidence of mechanical ventilation, we obtained the population data for China in 2022 from the China Statistical Yearbook 2023, published by the National Bureau of Statistics of China (https://www.stats.gov.cn/sj/ndsj/2023/indexeh.htm)

**Fig. 2:**
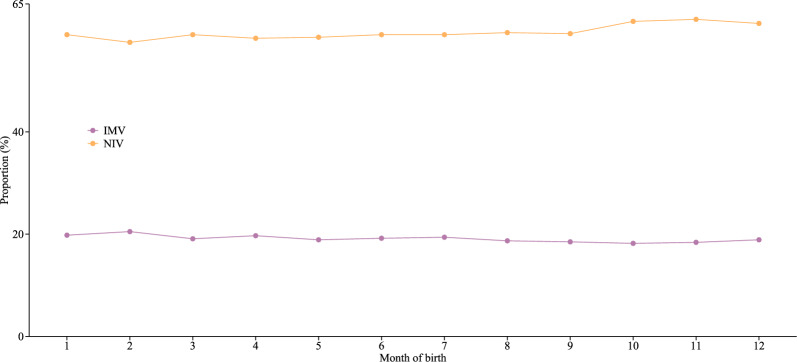
Distribution of invasive and non-invasive mechanical ventilation among neonates

Among the 149,153 newborns, the distribution of IMV and NIV across the twelve months was relatively balanced, but the proportion of NIV consistently exceeded that of IMV in each month (Fig. 2). The reason for the initiation of mechanical ventilation was heterogeneity and the primary diagnoses of patients requiring mechanical ventilation were respiratory failure (31.5%), pneumonia (24.2%), and sepsis (17.1%).

### Comorbidity

More than 50% of patients had a Charlson index of 2 or higher, particularly those requiring IMV (Table 1). The characteristics of individuals with comorbidities are shown in Table 2. Among the IMV cases, cerebrovascular disease was the most common comorbidity (41.4%), followed by congestive heart failure (36.7%) and diabetes without chronic complication (23.8%). Chronic pulmonary disease (57.0%), congestive heart failure (53.8%), and chronic obstructive pulmonary disease (30.2%) were frequently observed in patients receiving NIV.

**Table 2:** Distribution of comorbidities according to type of mechanical ventilation, sorted by frequency

Characteristics	IMV only (%)	NIV only (%)	Total population (%)
Comorbidity			
Yes	76.1	74.1	74.9
No	23.9	25.9	25.1
Individuals with comorbidity (N = 1 229 571)			
Age group, years			
0–6	1.33	2.2	1.9
6–18	0.8	0.2	0.6
18–65	42.1	24.0	36.0
65–80	39.0	46.5	41.4
> 80	16.9	27.1	20.1
Sex			
Male	64.8	67.0	65.5
Female	35.2	33.0	34.5
Type of comorbidity			
Congestive heart failure	36.7	53.8	42.7
Cerebrovascular disease	41.4	24.5	35.9
Chronic pulmonary disease	19.9	57.0	31.6
Diabetes without chronic complication	23.8	21.0	23.0
Chronic obstructive pulmonary disease	14.5	30.2	19.6
Renal disease	18.4	16.1	17.9
Mild liver disease	15.9	15.2	15.6
Peripheral vascular disease	11.1	14.0	12.0
Cancer	8.0	7.7	8.0
Peptic ulcer disease	6.4	2.1	5.1
Myocardial infarction	4.7	4.0	4.5
Diabetes with chronic complication	3.1	3.5	3.2
Dementia	2.2	3.0	2.4
Hemiplegia or paraplegia	2.2	0.6	1.7
Rheumatic disease	1.2	1.5	1.3

*IMV* invasive mechanical ventilation, *NIV* non-invasive mechanical ventilation

### Length of stay and case fatality rate

The median hospital stay from admission to discharge was 11.0 days (IQR: 6.0–20.0). Patients requiring NIV had a shorter median length of stay compared to those receiving IMV (10.0 days *vs* 12.0 days). Overall, 210,480 mechanically ventilated patients died during hospitalization, corresponding to an overall case fatality rate of 12.8%. The case fatality rate was 5.5% for patients receiving NIV only and 16.2% for those receiving IMV only (Table 1).

Discharge status stratified by age, sex, and comorbidity is shown in Table 3. A marked increase in case fatality rates was observed with advancing age, rising from 1.9% (95% CI: 1.8–2.0) among patients aged 0–6 years to 20.3% (95% CI: 20.2–20.5) in those aged over 80 years. The case fatality rate differed significantly between patients with different modes of ventilatory support across five age groups, with higher rates among those treated with IMV. Furthermore, males had a slightly higher case fatality rate compared to females (13.4% *vs.* 11.8%). The case fatality rate was also elevated among patients with a higher comorbidity burden, demonstrating a dose–response relationship between the Charlson index and mortality risk (Table 3).

**Table 3:** Case fatality rate stratified by age, sex, and comorbidity

	Case fatality rate, % (95% CI)			*P value*
	IMV only	NIV only	Total	
Age group, years				
0–6	5.7 (5.5–5.9)	0.3 (0.3–0.3)	1.9 (1.8–2.0)	< 0.001
6–18	13.1 (12.6–13.6)	3.5 (2.7–4.4)	12.0 (11.5–12.4)	< 0.001
18–65	14.0 (13.9–14.1)	4.2 (4.1–4.4)	12.3 (12.2–12.4)	< 0.001
65–80	16.7 (16.6–16.8)	5.1 (5.0–5.2)	13.1 (13.0–13.2)	< 0.001
80	25.1 (24.9–25.3)	12.2 (12.0–12.4)	20.3 (20.2–20.5)	< 0.001
Sex				
Male	17.1 (17.0–17.2)	5.5 (5.4–5.6)	13.4 (13.3–13.4)	< 0.001
Female	14.6 (14.5–14.7)	5.5 (5.3–5.6)	11.8 (11.7–11.9)	< 0.001
Comorbidity				
No	12.9 (12.8–13.1)	1.4 (1.4–1.5)	8.4 (8.3–8.4)	< 0.001
Yes	17.2 (17.1–17.3)	6.9 (6.8–7.0)	14.2 (14.2–14.3)	< 0.001
Patients with 1 Charlson index	13.8 (13.7–14.0)	3.7 (3.6–3.8)	11.2 (11.1–11.3)	< 0.001
Patients with 2 Charlson index	15.3 (15.1–15.5)	4.6 (4.4–4.7)	11.6 (11.4–11.7)	< 0.001
Patients with 3 Charlson index	17.2 (17.0–17.4)	6.3 (6.1–6.5)	13.7 (13.5–13.8)	< 0.001
Patients with 4 Charlson index	19.7 (19.4–20.0)	8.2 (7.9–8.5)	16.2 (16.0–16.4)	< 0.001
Patients with 5 Charlson index	23.7 (23.5–23.9)	14.5 (14.3–14.8)	21.0 (20.8–21.2)	< 0.001
Total	16.2 (16.1–16.3)	5.5 (5.4–5.5)	12.8 (12.8–12.9)	< 0.001

*IMV* invasive mechanical ventilation, *NIV* non-invasive mechanical ventilation, *CI* confidence interval

Among patients who received both modes of ventilatory support, a total of 40,619 experienced a progression from NIV to IMV. These patients represented 7.9% of the 516,684 patients who initially received NIV (Supplementary Table 4). Table 4 presents the baseline characteristics of patients who underwent a single transition between ventilatory strategies. Notably, patients who transitioned from NIV to IMV had a shorter hospital length of stay but a higher case fatality rate when compared to those who transitioned from IMV to NIV.

**Table 4:** Characteristics of patients who underwent a single transition between ventilatory modes

Characteristics	NIV transition to IMV	IMV transition to NIV
Number of admissions	36 206^*^	60 014^#^
Age, years		
Mean (SD)	57.8 (29.7)	44.7 (34.6)
Median (IQR)	69.0 (51.0–78.0)	59.0 (0.0–74.0)
Age group, years, n (%)		
0–6	6 356 (17.6)	20 629 (34.4)
6–18	237 (0.7)	514 (0.9)
18–65	8 818 (24.4)	13 383 (22.3)
65–80	13 577 (37.5)	17 599 (29.3)
> 80	7 218 (19.9)	7 889 (13.1)
Sex, n (%)		
Male	24 098 (66.6)	38 443 (64.1)
Female	12 105 (33.4)	21 571 (35.9)
Charlson index, n (%)		
0	7 758 (21.4)	22 280 (37.1)
1	6 225 (17.2)	10 593 (17.7)
2	6 478 (17.9)	8 670 (14.4)
≥ 3	15 745 (43.5)	18 471 (30.8)
Discharge status, n (%)		
Death	7 525 (20.8)	4 693 (7.8)
Survive	28 681 (79.2)	55 321 (92.2)
Length of hospital stay, days		
Mean (SD)	16.5 (19.5)	22.6 (21.2)
Median (IQR)	11.0 (6.0–20.0)	17.0 (10.0–28.0)

*IMV* invasive mechanical ventilation, *NIV* non-invasive mechanical ventilation, *SD* standard deviation, *IQR* Interquartile range

*A total of 4 413 admissions were excluded here due to complex transitions, such as switching from initial NIV to IMV and then back to NIV

^#^A total of 7 451 admissions were excluded here due to complex transitions, such as switching from initial IMV to NIV and then back to IMV

## Discussion

Although understanding the characteristics and distribution of mechanically ventilated patients is crucial for reducing the mortality risk in clinical practice, associated epidemiological data in China are limited. To improve the quality of healthcare programs and optimize the allocation of critical care resources, the intensive care departments, in collaboration with the National Health Commission, have established a comprehensive mechanical ventilation database. To the best of our knowledge, this is the first study to provide a detailed analysis of such a large-scale database on mechanical ventilation in China. Overall, the mean age of the study population was 59.2 years. Males accounted for a higher proportion of mechanically ventilated patients than females (64.5% *vs.* 35.5%). Patients receiving NIV were younger than those receiving IMV (57.3 *vs*. 61.2), with NIV being more frequently used in children and the elderly. Across all age, sex, and comorbidity groups, IMV was consistently associated with higher case fatality rates compared to NIV.

Our analysis revealed that patients over 80 years of age had a relatively lower percentage of IMV use compared to their younger counterparts. These findings align with studies conducted in the United States [15, 16], Argentina [17], and Canada [16, 18], which suggest that the intensity of treatment tended to decrease with advanced age [7]. Children under six years old, particularly neonates, prefer non-invasive options over invasive methods. This preference is primarily due to the non-invasive nature of NIV [19], which reduces the risks and complications associated with intubation, provides effective respiratory support, and achieves higher success rates in children. Furthermore, our study found that patients treated with IMV had a higher risk of mortality and longer hospital stays compared to those managed with NIV, possibly due to more severe underlying illness among patients requiring IMV.

Our study has important epidemiological and clinical implications. Despite the relatively small proportion of hospitalized patients requiring mechanical ventilation, this group often accounts for a significant share of healthcare resource consumption and contributes to rising medical costs [20]. Therefore, it is imperative to focus on reducing the prevalence of this group in the future. However, most low- and middle-income countries have little or no data to show ventilated patients’ characteristics or outcomes. Addressing this data gap is essential to identify local challenges, understand regional variations, implement evidence-based interventions, and improve outcomes for mechanically ventilated patients globally. As the first study to report the epidemiological characteristics of patients on mechanical ventilation in China, this study lays a crucial foundation for understanding the comprehensive profile of mechanical ventilation use, irrespective of NIV and IMV. The findings of this study underscore the heterogeneity of ventilated care and provide valuable information on potential high-risk groups with poor prognosis, such as the elderly, males, and individuals with comorbidities [21, 22], who are more likely to progress to severe illness or death. Early intervention in these vulnerable populations is strongly encouraged as a means to improve overall outcomes and reduce mortality risks. Our results also indicate that nearly half of the patients were from the Eastern regions. Furthermore, disparities in the distribution of healthcare resources, such as hospital beds and staff in intensive care, limit equitable access to mechanical ventilation services in China. The present study may offer valuable insights to guide the allocation of medical resources to underdeveloped areas. Lastly, our study emphasizes the importance of strengthening national administrative health data systems to enable more accurate, timely, and comprehensive monitoring of mechanical ventilation practices and patient outcomes.

### Strengths and limitations

This study represents the largest research in the use of mechanical ventilation across all 31 provinces in China. The extensive and detailed data allowed us to conduct multiple subgroup analyses. Nonetheless, despite its significance, our study is not without limitations. First, due to the significant heterogeneity and uneven development across different regions in China, we were unable to collect medical record data from all hospitals. Second, laboratory data and physiological indicators were not included in administrative electronic health records. The absence of these key parameters constrains our ability to evaluate the impact of physiological factors on ventilation outcomes. Future multicenter, hospital-based studies could help validate and provide a more comprehensive understanding. Lastly, as a specialized procedure, mechanical ventilation is highly dependent on medical resources and skilled personnel. Primary healthcare institutions, small clinics in rural areas, or hospitals from remote areas often lack the necessary equipment and qualified personnel for mechanical ventilation, therefore, we did not include these hospitals. Given their limited role in providing such specialized care, the results would not have a significant impact.

## Conclusions

In conclusion, the database presented here is the largest and most comprehensive contemporary dataset on mechanically ventilated patients admitted to hospitals in China. Based on this database, we have shown the epidemiological characteristics and outcomes of patients requiring different types of mechanical ventilation in China. Despite certain limitations, this dataset serves as a crucial foundation for future epidemiological studies and healthcare policy evaluations. Further efforts should focus on data validation, linkage with additional clinical records, and expanding the scope of analyses to enhance their utility in critical care research.

## Supplementary Information

Additional file1

## Data Availability

The datasets used and analyzed during the current study are available from the corresponding author on reasonable request.

## Abbreviations

NDCMS: National Data Center for Medical Service
NIV: Non-invasive ventilation
IMV: Invasive mechanical ventilation
ICD-9-CM: International Classification of Disease, Ninth Revision, Clinical Modification
ICD-10: International Classification of Diseases, Tenth Revision
SD: Standard deviation
IQR: Interquartile range
