# Supplementary material for: Population-wise incidence and outcomes of patients requiring invasive and non-invasive mechanical ventilation in China: a nationwide retrospective analysis by age, sex, and comorbidity
Source: Ann Intensive Care. 2025 Aug 14;15:120. doi: 10.1186/s13613-025-01537-w (PMC12354394; doi:10.1186/s13613-025-01537-w)
Supplement: Supplementary file 1 — Additional file1 [file 13613_2025_1537_MOESM1_ESM.docx]

| **Supplementary table 1. Main variable in the mechanical ventilation database** |
| --- |
| Unique identifier for each patient |
| Identifier of each hospital |
| Located province (or autonomous region, municipality) |
| Organization type |
| Hospital type |
| Gender |
| Age |
| Date of birth |
| Admission date |
| Admitting department |
| Admission route |
| Discharge date |
| Discharge department |
| Length of hospital stays |
| Diagnostic codes |
| Procedure codes |
| Date of procedure |
| Anesthesia method for surgical procedures |
| Age under 1 year (in days) |
| Birth weight |
| Rehospitalization within 31 days after discharge |
| Discharge type, including discharge due to in-hospital death |
| Total hospitalization cost |

**Supplementary Table 2. Definitions of comorbidity**

| Comorbidity | ICD-10-CM |
| --- | --- |
| Myocardial infarction | I21.x, I22.x, I25.2 |
| Congestive heart failure | I09.9, I11.0, I13.0, I13.2, I25.5, I42.0, I42.5 - I42.9, I43.x, I50.x, P29.0 |
| Peripheral vascular disease | I70.x, I71.x, I73.1, I73.8, I73.9, I77.1, I79.0, I79.2, K55.1, K55.8, K55.9, Z95.8, Z95.9 |
| Cerebrovascular disease | G45.x, G46.x, H34.0, I60.x - I69.x |
| Dementia | F00.x - F03.x, F05.1, G30.x, G31.1 |
| Chronic pulmonary disease | I27.8, I27.9, J40.x - J47.x, J60.x - J67.x, J68.4, J70.1, J70.3 |
| Rheumatic disease | M05.x, M06.x, M31.5, M32.x - M34.x, M35.1, M35.3, M36.0 |
| Peptic ulcer disease | K25.x - K28.x |
| Mild liver disease | B18.x, K70.0 - K70.3, K70.9, K71.3 - K71.5, K71.7, K73.x, K74.x, K76.0, K76.2 - K76.4, K76.8, K76.9, Z94.4 |
| Diabetes without chronic complication | E10.0, E10.1, E10.6, E10.8, E10.9, E11.0, E11.1, E11.6, E11.8, E11.9, E12.0, E12.1, E12.6, E12.8, E12.9, E13.0, E13.1, E13.6, E13.8, E13.9, E14.0, E14.1, E14.6, E14.8, E14.9 |
| Diabetes with chronic complication | E10.2 - E10.5, E10.7, E11.2 - E11.5, E11.7, E12.2 - E12.5, E12.7, E13.2 - E13.5, E13.7, E14.2 - E14.5, E14.7 |
| Hemiplegia or paraplegia | G04.1, G11.4, G80.1, G80.2, G81.x, G82.x, G83.0 - G83.4, G83.9 |
| Renal disease | I12.0, I13.1, N03.2 - N03.7, N05.2 - N05.7, N18.x, N19.x, N25.0, Z49.0 - Z49.2, Z94.0, Z99.2 |
| Any malignancy, including lymphoma and leukaemia, except malignant neoplasm of skin | C00.x - C26.x, C30.x - C34.x, C37.x - C41.x, C43.x, C45.x - C58.x, C60.x - C76.x, C81.x - C85.x, C88.x, C90.x - C97.x |
| Moderate or severe liver disease | I85.0, I85.9, I86.4, I98.2, K70.4, K71.1, K72.1, K72.9, K76.5, K76.6, K76.7 |
| Metastatic solid tumour | C77.x - C80.x |
| AIDS/HIV | B20.x - B22.x, B24.x |

Charlson ME, Pompei P, Ales KL, et al. A new method of classifying prognostic comorbidity in longitudinal studies: development and validation. Journal of Chronic Diseases 1987; 40:373-383. DOI: 10.1016/0021-9681(87)90171-8

Sharabiani MT, Aylin P, Bottle A. Systematic review of comorbidity indices for administrative data. Medical Care 2012; 50(12):1109-1118. DOI: 10.1097/MLR.0b013e31825f64d0

ICD-10=International Classification of Diseases 10th revision

| **Supplementary Table 3. Baseline characteristics of patients requiring mechanical ventilation stratified by regions** | | | | | | |
| --- | --- | --- | --- | --- | --- | --- |
| Characteristics | East | | Central | | West | |
|  | IMV | NIV | IMV | NIV | IMV | NIV |
| Number of admissions | 488 869 | 187 322 | 264 184 | 111 389 | 304 607 | 177 354 |
| Number of hospitals, n (%) | 1167 | 1100 | 725 | 685 | 905 | 875 |
| Age, years |  |  |  |  |  |  |
| Mean (SD) | 62.3 (20.4) | 58.9 (30.9) | 61.1 (20.3) | 56.1 (31.2) | 59.6 (20.8) | 56.4 (30.8) |
| Median (IQR) | 66.0 (53.0-76.0) | 70.0 (52.0-80.0) | 65.0 (53.0-75.0) | 69.0 (52.0-76.0) | 64.0 (51.0-74.0) | 68.0 (48.0-78.0) |
| Age group, years, n (%) |  |  |  |  |  |  |
| 0-6 | 18 792 (3.8) | 35 126 (18.8) | 11 565 (4.4) | 23 324 (20.9) | 13 897 (4.6) | 35492 (20.0) |
| 6-18 | 6 977 (1.4) | 703 (0.4) | 4 314 (1.6) | 396 (0.4) | 5 737 (1.9) | 831 (0.5) |
| 18-65 | 210 034 (43.0) | 36 530 (19.5) | 117 422 (44.4) | 24 252 (21.8) | 144 399 (47.4) | 40652 (22.9) |
| 65-80 | 173 212 (35.4) | 69 124 (36.9) | 95 935 (36.3) | 42 259 (37.9) | 102 146 (33.5) | 66422 (37.5) |
| >80 | 79 854 (16.3) | 45 839 (24.5) | 34 948 (13.2) | 21 158 (19.0) | 38 428 (12.6) | 33957 (19.1) |
| Sex, n (%) |  |  |  |  |  |  |
| Male | 315 501 (64.5) | 123 131 (65.7) | 168 256 (63.7) | 74 819 (67.2) | 192 583 (63.2) | 115 066 (64.9) |
| Female | 173 365 (35.5) | 64 191 (34.3) | 95 928 (36.3) | 36 570 (32.8) | 112 024 (83.2) | 62 288 (35.1) |
| Discharge status, n (%) |  |  |  |  |  |  |
| Death | 77 282 (15.8) | 11 555 (6.2) | 42 947 (16.3) | 5 057 (4.5) | 51 033 (16.8) | 9 409 (5.3) |
| Survive | 411 587 (84.2) | 175 767 (93.8) | 221 237 (83.7) | 106 332 (95.5) | 253 574 (83.2) | 167 945 (94.7) |
| Length of hospital stay, days |  |  |  |  |  |  |
| Mean (SD) | 18.2 (23.1) | 13.8 (13.8) | 15.1 (19.4) | 13.0 (12.0) | 16.0 (20.2) | 12.1 (10.4) |
| Median (IQR) | 13.0 (5.0-23.0) | 10.0 (7.0-16.0) | 10.0 (3.0-20.0) | 10.0 (7.0-15.0) | 11.0 (4.0-21.0) | 10.0 (7.0-14.0) |
| Charlson index, n (%) |  |  |  |  |  |  |
| 0 | 117 917 (24.1) | 48 763 (26.0) | 62 671 (23.7) | 29 247 (26.3) | 72 192 (23.7) | 45 398 (25.6) |
| 1 | 117 966 (24.1) | 34 418 (18.4) | 66 260 (25.1) | 23 580 (21.2) | 73 097 (24.0) | 34 177 (19.3) |
| 2 | 84 193 (17.2) | 37 184 (19.9) | 46 686 (17.7) | 23 219 (20.8) | 49 883 (16.4) | 36 466 (20.6) |
| ≥3 | 168 793 (34.5) | 66 957 (35.7) | 88 567 (33.5) | 35 343 (31.7) | 109 435 (35.9) | 61 313 (34.6) |
| Initiation reasons, n (%) |  |  |  |  |  |  |
| Respiratory failure | 114987 (45.1) | 79 509 (35.4) | 72 203 (28.3) | 54 297 (24.2) | 68 015 (26.7) | 90 974 (40.5) |
| Pneumonia | 98 204 (44.9) | 51 741 (36.4) | 51 373 (23.5) | 29 020 (20.4) | 69 318 (31.7) | 61 441 (43.2) |
| Sepsis | 80 826 (41.1) | 20 040 (33.5) | 50 801 (25.8) | 14 335 (24.0) | 65 097 (33.1) | 25 467 (42.6) |
| IMV: invasive mechanical ventilation; NIV: non-invasive mechanical ventilation; SD: standard deviation; IQR: Interquartile range.  East: Beijing, Tianjin, Hebei, Liaoning, Shanghai, Jiangsu, Zhejiang, Fujian, Shandong, Guangdong, and Hainan.  Central: Shanxi, Jilin, Heilongjiang, Anhui, Jiangxi, Henan, Hubei, and Hunan.  West: Inner Mongolia, Chongqing, Guangxi, Sichuan, Guizhou, Yunnan, Tibet, Shaanxi, Gansu, Qinghai, Ningxia, and Xinjiang. | | | | | | |

| **Supplementary Table 4. Baseline characteristics of patients who initially required NIV** | | | |
| --- | --- | --- | --- |
| Characteristics | NIV  initiation | No transition | NIV failure |
| Number of admissions | 516 684 | 476 065 | 40 619 |
| Age, years |  |  |  |
| Mean (SD) | 57.2 (31.0) | 57.3 (30.9) | 55.4 (31.2) |
| Median (IQR) | 69.0 (49.0-79.0) | 69.0 (50.0-79.0) | 68.0 (44.0-78.0) |
| Age group, years, n (%) |  |  |  |
| 0-6 | 102 441 (19.8) | 93 942 (19.7) | 8 499 (20.9) |
| 6-18 | 2 209 (0.4) | 1 930 (0.4) | 279 (0.7) |
| 18-65 | 110 874 (21.5) | 101 434 (21.3) | 9 440 (23.2) |
| 65-80 | 192 475 (37.3) | 177 805 (37.4) | 14 670 (36.1) |
| >80 | 108 685 (21.0) | 100 954 (21.2) | 7 731 (19.1) |
| Sex, n (%) |  |  |  |
| Male | 339 992 (65.8) | 313 016 (65.8) | 26 976 (66.4) |
| Female | 176 692 (34.2) | 163 049 (34.2) | 13 643 (33.6) |
| Charlson index, n (%) |  |  |  |
| 0 | 133 201 (25.8) | 123 408 (25.9) | 9 793 (24.0) |
| 1 | 98 984 (19.2) | 92 175 (19.4) | 6 809 (16.8) |
| 2 | 103 884 (20.0) | 96 869 (20.3) | 7 015 (17.3) |
| ≥3 | 180 615 (35.0) | 163 613 (34.4) | 17 002 (41.9) |
| Discharge status, n (%) |  |  |  |
| Death | 34 053 (6.6) | 26 021 (5.5) | 8 032 (19.8) |
| Survive | 482 631 (93.4) | 450 044 (94.5) | 32 587 (80.2) |
| Length of hospital stay, days |  |  |  |
| Mean (SD) | 13.3 (13.1) | 13.0 (12.2) | 17.6 (20.3) |
| Median (IQR) | 10.0 (7.0-16.0) | 10.0 (7.0-15.0) | 12.0 (6.0-22.0) |
| NIV: non-invasive mechanical ventilation; SD: standard deviation; IQR: Interquartile range. | | | |
